# Mothers Who Work

**Published:** 1920-01-24

**Authors:** 


### Mothers Who Work.

The theory that infantile mortality is highest in cases where mothers go out to work is not supported by Dr. Sidney Barwise, the medical officer of health for Derbyshire. In his annual report he says it is interesting to note that in Derbyshire the districts with the high infantile mortality are not those where a large proportion of the women go out to work. The predominating occupation in the districts of high infantile mortality is that of coal mining. As a class the coal miners, compared with other workers, are well off, but their wives, on whom the health of the children largely depends, have in the past received no instruction in home management, though even given all possible instructions in this respect it is impossible to bring up healthy children in a house with an unpaved common yard and a privy-midden. Infantile mortality will be reduced when the homes of the people are such that the women may be proud of them.

